# Acceptance of pharmacist-led stewardship recommendations for patients with community-acquired pneumonia

**DOI:** 10.1017/ash.2024.399

**Published:** 2024-10-17

**Authors:** Ramara E. Walker, Rebecca Schulte, Andrea M. Pallotta, Larisa G. Tereshchenko, Victoria A. Criswell, Abhishek Deshpande, Michael B. Rothberg

**Affiliations:** 1 Department of Pharmacy, Cleveland Clinic, Cleveland, OH, USA; 2 Department of Quantitative Health Sciences, Cleveland Clinic, Cleveland, OH, USA; 3 Center for Value-based Care Research, Primary Care Institute, Cleveland Clinic, Cleveland, OH, USA; 4 Department of Infectious Disease, Cleveland Clinic, Cleveland, OH, USA

## Abstract

**Background:**

Community-acquired pneumonia (CAP) is a leading cause of hospitalizations and mortality in the US. Overuse of extended spectrum antibiotics (ESA) for CAP contributes to antimicrobial resistance. The 2019 Infectious Diseases Society of America/American Thoracic Society CAP guidelines emphasize de-escalation of ESA following negative cultures, early switch to oral (PO) antibiotics, and limited duration of therapy (DOT). This study describes clinicians’ acceptance of an infectious diseases-trained (ID) pharmacist-led stewardship recommendations in hospitalized patients with CAP.

**Methods:**

This prospective, single-arm, cohort study included adults admitted with a diagnosis of pneumonia to six Cleveland Clinic hospitals receiving ID pharmacist-led stewardship recommendations. The ID pharmacist provided recommendations for ESA de-escalation, DOT, intravenous (IV) to PO transition, and antimicrobial discontinuation. Descriptive statistics were used to describe clinician acceptance rates.

**Results:**

From November 1, 2022, to January 31, 2024, the ID pharmacist made recommendations for 685 patient encounters to 327 clinicians. Of these patients, 52% received an ESA and 15% had severe CAP. There were 959 recommendations: ESA de-escalation (19%), DOT (46%), IV to PO transition (19%), antimicrobial discontinuation (13%), and other (3%). Clinicians accepted 693 recommendations (72%): IV to PO transition (148/184, 80%), ESA de-escalation (141/181 78%), antimicrobial discontinuation (94/128, 73%), DOT (286/437, 65%), and other (24/29, 83%).

**Conclusion:**

Clinicians were generally receptive to ID pharmacist-led CAP recommendations with an overall acceptance rate of 72%. Prescribers were most receptive to recommendations for IV to PO conversion and least receptive to limiting DOT.

## Introduction

Community-acquired pneumonia (CAP) is one of the most common causes of hospitalizations and mortality in the United States.^1^ Historically, targeted treatment of CAP has been difficult due to low rates of pathogen detection.^2^ As a result, many patients with CAP receive empiric extended-spectrum antimicrobial therapy (ESA), which could lead to unnecessary adverse effects and antimicrobial resistance. Additionally, the previous practice designating patients with CAP as having health-care associated pneumonia (HCAP) based on risk factors for hospital-associated pathogens such as methicillin-resistant *Staphylococcus aureus* and *Pseudomonas aeruginosa* has led to widespread use of ESAs for CAP, despite the removal of the HCAP designation from pneumonia treatment recommendations from Infectious Diseases Society of America (IDSA)/American Thoracic Society (ATS).^3^ The 2019 IDSA/ATS CAP guidelines also emphasize de-escalation of ESA following negative cultures, early switch to oral (PO) antibiotics, and limited DOT guided by a validated measure of patient clinical stability criteria.^4^

Previous studies evaluated the impact of pharmacist-led antimicrobial stewardship (ASP) interventions on hospitalized patients with various infectious syndromes including CAP and demonstrated that pharmacist-led ASP interventions pertaining to drug selection, dosage, route of administration, DOT, and de-escalation can safely reduce unnecessary antimicrobial use without increasing mortality.^5,6^ These studies have included bundled intervention strategies (e.g., direct, prospective audit and feedback, academic detailing, educational presentation and feedback sessions, letters to prescribers, wall posters, and prescribing prompts) increasing the likelihood of physician acceptance.^7,8^ Less is known about physicians’ receptivity to specific types of pharmacist recommendations for CAP in the course of usual care. The aim of this study was to describe clinician acceptance of infectious diseases pharmacist-led ASP recommendations in hospitalized patients with CAP across a large US healthcare system.

## Methods

We conducted a prospective, single-arm cohort study that included patients ≥18 years of age who were admitted with a diagnosis of pneumonia to six Cleveland Clinic Health System hospitals which received ID pharmacist-led CAP recommendations from November 1, 2022, to January 31, 2024. Although a patient may have been admitted multiple times during the study period, we considered each admission as an independent observation. Two of the six hospitals were classified as teaching hospitals. There were no patient exclusion criteria. One 0.5 FTE ID trained and board-certified pharmacist remotely evaluated patients’ charts daily on weekdays and assessed patients for clinical stability. The ID pharmacist received and reviewed an automated daily list of eligible patients who met inclusion and clinical stability criteria. Communication with clinicians occurred via secure chat, phone, or page. All interventions were documented prospectively within an Oracle database. Clinical stability was defined according to the 2019 IDSA/ATS CAP Guidelines as having a body temperature of 38°C or less for 48 hours and having no more than 1 CAP-associated sign of clinical instability (systolic blood pressure less than 90 mm Hg, heart rate greater than 100 beats/minute, respiratory rate greater than 24 breaths/minute, and arterial oxygen saturation less than 90% or PaO_2_ less than 60 mm Hg in room air).^4^ Patients on supplemental oxygen prior to hospitalization were considered stable from a respiratory standpoint if they were able to be successfully weaned back to their home oxygen requirements. In addition to meeting clinical stability criteria, patients required negative blood cultures for 48 hours and/or respiratory cultures demonstrating an organism sensitive to non-ESA therapy (i.e., ampicillin/sulbactam, amoxicillin/clavulanate, ceftriaxone, cefdinir, cefuroxime, cefpodoxime, azithromycin, doxycycline, levofloxacin, and moxifloxacin) to qualify for an ID pharmacist recommendation. This study defined ESA as the following: piperacillin-tazobactam, aztreonam, imipenem-cilastatin, meropenem, ertapenem, cefepime, ceftazidime, tobramycin, linezolid, vancomycin, ceftazidime-avibactam, ceftolozane-tazobactam, meropenem-vaborbactam, imipenem-cilastatin-relebactam, cefiderocol, ceftaroline, tigecycline, eravacycline, and amikacin. This study was approved by Cleveland Clinic’s Institutional Review Board.

### Antimicrobial stewardship program

Antimicrobial stewardship at Cleveland Clinic includes dissemination of treatment guidelines, including CAP, via an intranet web application accessible on all Cleveland Clinic computers and issued devices. Additionally, a CAP order set is available for clinicians upon admission, but our sepsis order set is used more commonly, which includes recommendations for CAP with and without risk factors for multidrug resistant pathogens. The preferred CAP treatment is ceftriaxone plus azithromycin for 5 days with levofloxacin as the alternative for patients with severe beta-lactam allergies. Vancomycin plus *S. aureus* nasal polymerase chain reaction (PCR) testing and/or piperacillin/tazobactam are considered for patients with prior respiratory infection with methicillin-resistant *Staphylococcus aureus* or *Pseudomonas aeruginosa* within one year or hospitalization with receipt of parenteral antibiotics within 90 days. Stop dates are only included on the CAP order set. Providers select one of three indications on all antimicrobial orders: empiric, pathogen-directed, prophylaxis. The intervening pharmacist utilized these documents when contacting clinicians. Our institution will soon implement mandatory syndrome-specific indications (i.e.: respiratory, urinary, and skin/soft tissue) on all antimicrobial orders, which can assist with targeted DOT recommendations using ASP alerts. Lastly, the study pharmacist provided email education on CAP DOT to hospital-site champions to disseminate to clinicians at each of the hospitals.

### Measures

The primary outcome was whether the ID pharmacist’s recommendations were accepted. Clinicians included physicians and advance practice practitioners (APPs) such as nurse practitioners and physician assistants. Secondary outcomes included the types of recommendations attempted; acceptance rates based on hospital, month and day of recommendation, and clinician demographics; and reasons for intervention non-acceptance.

Demographic and clinical baseline characteristics of patients were also collected.

### Recommendations

The ID pharmacist’s recommendation types included the following: (1) gram-positive de-escalation defined as de-escalation of an anti-MRSA agent to targeted CAP therapy [targeted CAP therapy was defined as combination therapy with ampicillin-sulbactam or ceftriaxone (or oral equivalents) plus azithromycin or doxycycline. Monotherapy with a respiratory fluoroquinolone was also considered guideline concordant per the 2019 IDSA/ATS CAP guidelines.^4^], (2) gram-negative de-escalation defined as de-escalation of an anti-pseudomonal agent to targeted CAP therapy, (3) non-atypical CAP agent discontinuation in response to a positive microbiological result for an atypical organism such as *Legionella pneumophila*, (4) atypical CAP agent discontinuation in response to a positive microbiological result for a non-atypical organism such as *Streptococcus pneumoniae*, (5) antibiotic discontinuation in response to a positive microbiological result indicating a non-bacterial infection in which all antibiotics prescribed were recommended to be discontinued, (6) IV to PO transition of atypical CAP agent and/or non-atypical CAP agent if clinically stable and able to tolerate oral medications as evidenced by administration records within the electronic medical record (EMR), (7) DOT (including discharge antibiotic days if applicable) – 5 days, 6–7 days, or >7 days was defined as the number of days of antibiotic therapy from initiation to discontinuation, and (8) “other” which was used as a catch-all.

### Statistical analysis

Normally distributed variables were summarized as mean and standard deviation (SD). Non-normally distributed variables were summarized as a median and interquartile range (IQR). Categorical variables were summarized as frequency counts. The number of recommendations and rate of acceptance were compared across recommendation types, hospital, clinician type, and month and day of the week, using Pearson’s chi-squared statistic.

Analyses were conducted using R version 4.2.1.

## Results

From November 1, 2022, to January 31, 2024, the ID pharmacist made 959 antibiotic recommendations to 327 clinicians regarding 685 patient encounters for an average of 1.4 recommendations per patient. The overwhelming majority of communications were via EMR secure chat. Clinicians were generally contacted once per recommendation. Only 2.5% of recommendations were repeated due to no response for 24 hours.

Table 1 describes the patient encounters about whom at least one recommendation was delivered. The mean age was 71 years, 51% were female, 69% were white, and 15% had a CURB65 (confusion, uremia, respiratory rate, blood pressure, and age ≥ 65 years) score of three or higher at admission. Fifty-two percent of patient encounters received an ESA. Consults to ID and Pulmonary were placed in 37% and 35% of patient encounters, respectively. The ID pharmacist was more likely to make a recommendation when pulmonary was consulted compared to when ID was consulted (43% vs 28%, *P* < 0.001). Patients’ baseline characteristics were evaluated for all encounters and the first encounter per patient to assess for any statistical differences. Analysis of each patient’s first encounter found similar results (e-Table1).

**Table 1.** Baseline characteristics of patients admitted with community-acquired pneumonia

Characteristic	Patient characteristics based on recommendation delivered			
	Overall (n = 5,964)	No Recommendation (n = 5,279)	Recommendation (n = 685)	*P* value
Age, years	71 (16)	71 (16)	73 (15)	0.002
Gender				0.7
Female	3,016 (51%)	2,675 (51%)	341 (50%)	
Male	2,948 (49%)	2,604 (49%)	344 (50%)	
Ethnic Group				0.4
African American	1,544 (26%)	1,361 (26%)	183 (27%)	
Asian/Pacific Islander	55 (0.9%)	49 (0.9%)	6 (0.9%)	
White	4,102 (69%)	3,628 (69%)	474 (69%)	
Hispanic	127 (2.1%)	115 (2.2%)	12 (1.8%)	
Multi-Racial	58 (1.0%)	50 (0.9%)	8 (1.2%)	
Native American	7 (0.1%)	7 (0.1%)	0 (0%)	
Other	53 (0.9%)	52 (1.0%)	1 (0.1%)	
Patient Refused	18 (0.3%)	17 (0.3%)	1 (0.1%)	
Extended Spectrum Antibiotics	3,101 (52%)	2,721 (52%)	380 (55%)	0.053
CURB65 score	1 (1)	1 (1)	2 (1)	0.085
Severe CAP	912 (15%)	800 (15%)	112 (16%)	0.4
Admission to ICU	1,046 (18%)	997 (19%)	49 (7.2%)	<0.001
Vasopressors	466 (7.8%)	443 (8.4%)	23 (3.4%)	<0.001
MRSA Swab	3,085 (52%)	2,716 (51%)	369 (54%)	0.2
Positive MRSA Swab	310 (10%)	292 (11%)	18 (4.9%)	<0.001
Pneumococcal Vaccine	4,623 (78%)	4,079 (77%)	544 (79%)	0.2
Respiratory Virus Panel	702 (12%)	606 (11%)	96 (14%)	0.053
Positive Respiratory Virus Panel	186 (26%)	158 (26%)	28 (29%)	0.5
Respiratory Culture	1,426 (24%)	1,227 (23%)	199 (29%)	<0.001
Positive Respiratory Culture	463 (32%)	403 (33%)	60 (30%)	0.5
Blood Culture	3,791 (64%)	3,333 (63%)	458 (67%)	0.057
Positive Blood Culture	474 (13%)	435 (13%)	39 (8.5%)	0.006
Length of Stay, days	5.8 (5.3)	5.7 (5.4)	6.8 (4.9)	<0.001
Consult to Infectious Disease	2,192 (37%)	2,002 (38%)	190 (28%)	<0.001
Consult to Pulmonary Medicine	2,090 (35%)	1,794 (34%)	296 (43%)	<0.001

a Patient characteristic data are represented at the patient encounter level.

b Values are presented as n (%) or mean (SD) unless otherwise stated.

3 Pearson’s Chi-squared test; Wilcoxon Rank Sum Test; Fisher’s Exact Test.

**Figure 1.**
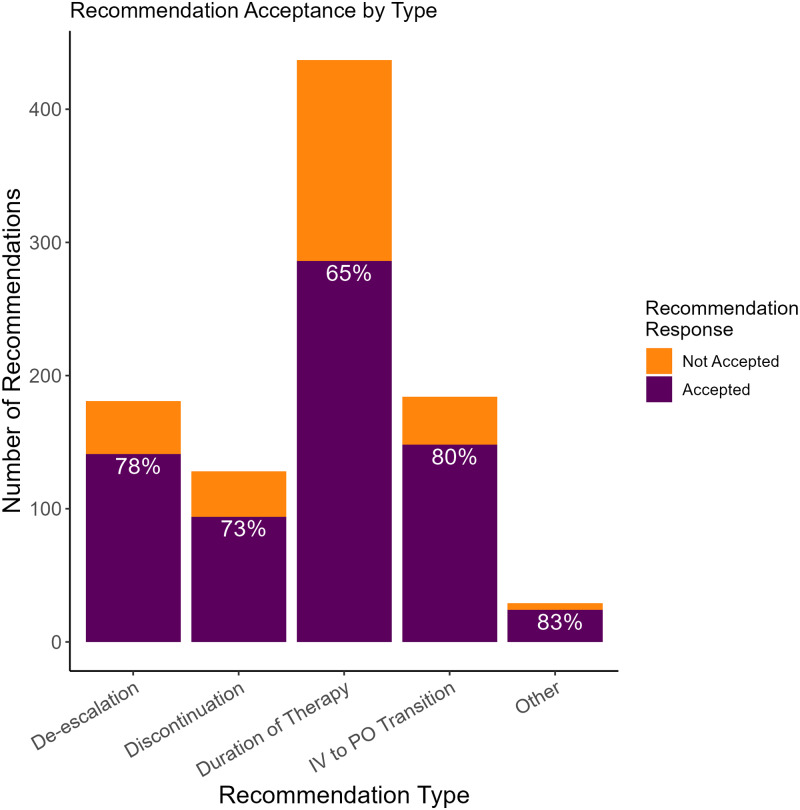
Pharmacist recommendation acceptance by recommendation type. Figure 1 displays the acceptance of pharmacist recommendations for Community-Acquired Pneumonia (CAP) treatment by recommendation type. Each bar represents the number of recommendations made, subdivided into accepted (purple) and not accepted (orange) categories.

**Figure 2.**
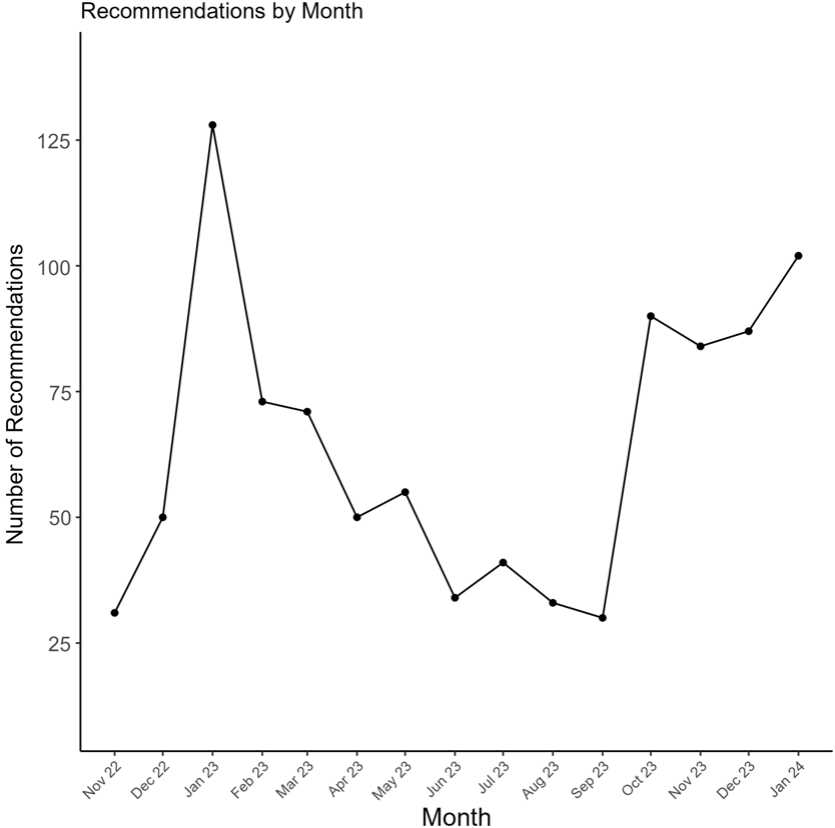
Trends in pharmacist recommendation numbers by month. Figure 2 depicts the number of pharmacist-led recommendations for patients with Community-Acquired Pneumonia (CAP) by month, from November 2022 to January 2024. Each point on the line graph represents the total number of recommendations made within a given month across the Cleveland Clinic Health System hospitals.

**Figure 3.**
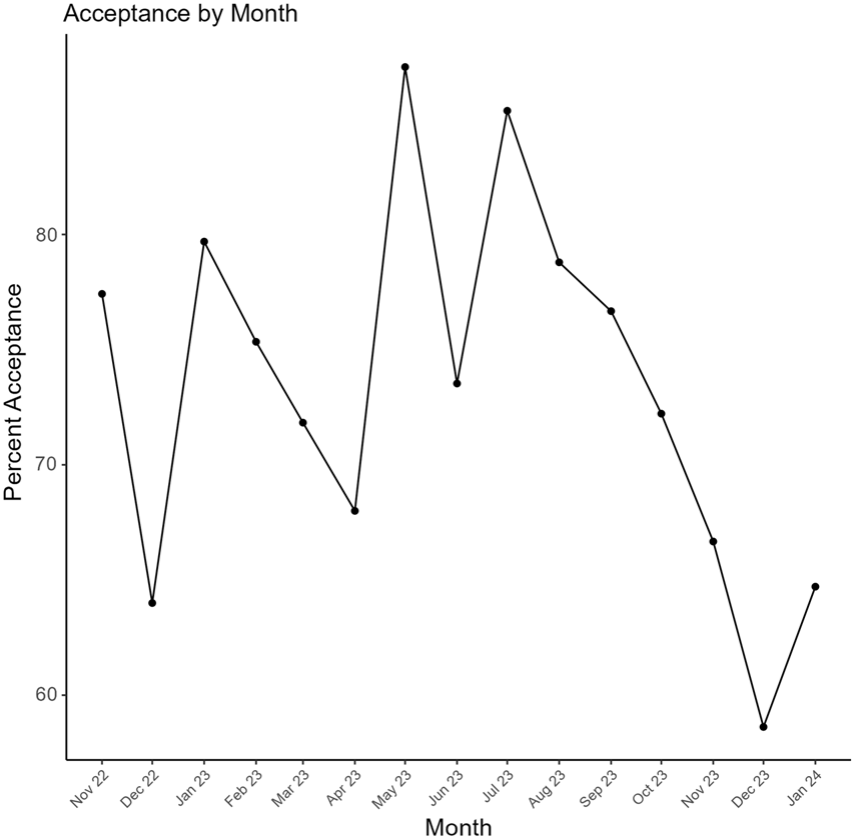
Monthly acceptance rates for pharmacist recommendations. Figure 3 presents the monthly acceptance rates of pharmacist recommendations from November 2022 to January 2024. Each data point represents the percentage of recommendations accepted by clinicians within that month.

Of the 959 recommendations that were delivered to clinicians, 693 (72%) were accepted. The acceptance rate for recommendations varied from 65% to 83% across types (*P* < 0.001) (Figure 1). For DOT recommendations, durations of 5 days were less accepted than durations of 6–7 days or 7+ days (58% vs 70% vs 88%, *P* = 0.005). Acceptance varied across hospitals in total (Table 2) (*P* = 0.02). Recommendations peaked in January and then decreased substantially during the spring and summer months (Figure 2). Acceptance ranged from 57% to 89% throughout the study period (*P* = 0.003) (Figure 3). The ID pharmacist made the most recommendations on Mondays and Tuesdays (n = 233 and 242, respectively) with the highest acceptance rates on Wednesdays and Thursdays (74%).

**Table 2.** Pharmacist recommendation acceptance by hospital and recommendation type

Recommendation type	Hospital 1 (%)	Hospital 2 (%)	Hospital 3 (%)	Hospital 4 (%)	Hospital 5 (%)	Hospital 6 (%)	*P* value
De-escalation	88.9	88.2	66.1	83	81.8	75	0.15
Discontinuation	84.2	88.9	63.6	73.1	74.1	56.3	0.23
Duration of Therapy	71.7	64.3	66.7	65.5	58.1	71.1	0.55
IV to PO Transition	89.7	91.7	79.5	79.3	73.7	60	0.10
Other	87.5	100	60	75	100	N/A	0.41
**Total**	**81.3**	**78.2**	**68.3**	**73.4**	**67.4**	**67.1**	**0.02**

Percentages represent the proportion of recommendations accepted by clinicians in each hospital. The ‘Other’ category includes various recommendations not classified under the primary types listed. ‘N/A’ indicates data was not available for that category at Hospital 6. The *P* values assess the statistical significance of the variation in acceptance rates across hospitals for each recommendation type.

One hundred and fifty clinicians received at least three recommendations. Of those, physicians and APPs had similar rates of acceptance (70% vs 75%, respectively, *P* = 0.36) (Table 3). Forty-one (27%) clinicians had an acceptance rate of 100%. The top three reasons for non-acceptance of recommendations were persistent CAP symptoms, clinician clinical judgment, and immunocompromising condition.

**Table 3.** Acceptance of pharmacist recommendations by provider type

Recommendation type	APP Acceptance (%) (n = 270)	Physician acceptance (%) (n = 566)	*P* value
De-escalation	81	74	0.50
Discontinuation	77	71	0.69
Duration of Therapy	69	64	0.40
IV to PO Transition	85	80	0.53
Other	60	90	0.34
**Total**	**75**	**71**	**0.36**

Percentages represent the proportion of each type of recommendation accepted by Advanced Practice Providers (APPs) and Physicians. *P* values are calculated to determine the statistical significance of the difference in acceptance rates between provider types, with a value less than 0.05 generally considered significant.

## Discussion

This study assessed the acceptance of an ID pharmacist-led ASP recommendations to clinicians for CAP inpatients and demonstrated high acceptance of recommendations overall (72%). The ID pharmacist prioritized making gram-positive and gram-negative de-escalation recommendations followed by DOT and IV to PO recommendations. Overall, the ID pharmacist impacted 11.5% of patients enrolled.

Acceptance rates varied depending on recommendation type. Recommendations for ESA de-escalation and IV to PO transition were most accepted while fewer clinicians accepted recommendations regarding limiting DOT, especially to 5 days. This is important because DOT recommendations were the most frequent recommendation made by the ID pharmacist (437/959, 46%), yet they had the lowest acceptance rate (65%). Acceptance rates did not differ based on clinician type (physician vs APP). The top reason for non-acceptance of the ID pharmacist’s recommendations was persistent CAP symptoms. Multiple studies have demonstrated that the resolution of CAP symptoms varies depending upon several patient-specific factors including age,^9^ comorbid conditions particularly chronic obstructive pulmonary disease,^10^ active smokers,^11^ and the infecting pathogen.^12^ Wootton and colleagues evaluated the average time to CAP recovery in 169 patients using a CAP symptom questionnaire and found that most patients returned to their pre-pneumonia baseline with 97% of CAP symptoms resolving within 10 days.^13^ With data demonstrating that patients’ symptoms may persist even after appropriate CAP treatment, it is important to discuss with clinicians which CAP symptoms are expected to readily resolve (e.g., fever) versus those that may resolve gradually over time (e.g., cough, chest pain, shortness of breath, and fatigue).^14^

Previous CAP studies demonstrated that shorter antibiotic courses are noninferior^15^ and have similar clinical success rates^16–20^ to longer ones. Hence, the most recent IDSA/ATS CAP Guidelines recommend a minimum 5-day antibiotic course for CAP. Nevertheless, we found that clinicians were hesitant to prescribe short antibiotic courses, even for patients meeting clinical stability criteria. Additionally, multiple studies have previously shown successful CAP DOT reduction by implementing ASP, educational, and statewide quality initiative efforts related to CAP.^21–23^ Our study demonstrated that clinician DOT acceptance increased as length of days recommended increased. Almost all the ID pharmacist’s recommendations for DOT were made for 5-7 days of therapy, which is consistent with current IDSA/ATS CAP guideline recommendations.

Moreover, this study included immunocompromised patients, which have been excluded from the most recent IDSA/ATS CAP guidelines.^4^ Given this, a CAP treatment consensus statement in immunocompromised adult patients was published in 2020 by a multidisciplinary panel of 45 physicians; however, it did not include recommendations for optimal DOT.^24^ Immunocompromised patients may require prolonged antibiotic courses depending upon the severity of immunosuppression.^7,25^ The tentativeness to embrace shorter DOT may be influenced by the current IDSA/ATS CAP guidelines that recommend a minimum 5-day DOT without explicit parameters offered for when longer courses are appropriate.

Our IV to PO recommendations were accepted the most. Previous studies have determined that IV to PO antibiotic conversion was safe and associated with shorter length of stay and fewer total antibiotic days.^26,27^ In one observational pre-post intervention study of inpatient CAP, IV to PO antibiotic conversion rates increased from 68% to 97% with the implementation of computerized reminders in the EMR.^27^ Patient factors associated with fewer IV to PO conversions included the presence of comorbidities, older age, secondary infection, and the patient’s rejection of oral medications whereas physician factors included physicians’ doubts about oral antibiotics and opinion of supervisor. Similarly in our study, the top three reasons for non-acceptance of IV to PO recommendations included patient clinical stability, persistent CAP symptoms, and immunocompromising condition.

Although our study demonstrated high initial ESA use overall, clinicians were generally receptive to de-escalating ESAs. The CAP-PACT trial evaluated whether an antibiotic stewardship intervention would reduce ESA use in moderately severe CAP in the Netherlands.^28^ The intervention incorporated education, engagement of local opinion leaders, and prospective audit and feedback of antibiotic use. Of 330 recommendations to switch antibiotics, 197 (59.7%) were accepted, considerably lower than was seen in our study. Differences may reflect the location, severity of illness, recommendations presented after microbiology and clinical stability data were confirmed, and timing of the study.

Even though pneumonia can occur any time of the year, we found that pharmacist recommendations peaked during respiratory season and declined during the spring and summer months, which suggests that dedicated ID pharmacist time should be maximized during respiratory season and can be reduced during the off-season. Notably, acceptance rates were lowest particularly during December 2022 and 2023 and highest during May and July 2023. It can be inferred that clinicians were more hesitant to accept recommendations for patients with CAP during the middle of respiratory illness season. The study pharmacist observed clinicians’ preference to maintain patients on ESA therapy until discharge even though patients were discharged home on CAP-targeted therapy. This trend occurred especially during the winter months despite the study pharmacist’s efforts. Lastly, for systems with limited resources, effort may be targeted early in the week given that our study pharmacist made the most recommendations on Tuesdays and the least on Fridays.

A notable strength of this study is its conduct under real-world conditions. Hundreds of autonomous clinicians at six different hospitals were able to openly accept or reject recommendations, providing an estimate of how acceptable such recommendations are likely to be in practice. This study also incorporated various hospital and clinician types (e.g., large, academic medical center, small community hospitals, and hospital-based vs. private-practicing clinicians) within the health system thereby increasing the sample population and overall generalizability of these results. The variation of recommendation acceptance rates across clinicians warrants further exploration as some clinicians accept or reject all recommendations independent of the recommendation category.

There were several limitations to our study. First, only one part-time (0.5 FTE) ID pharmacist performed daily chart review, excluding weekends, and offered recommendations to clinicians, which may have introduced investigator bias. This limitation affected the volume of recommendations made for eligible patients as well as the interpretation of our results. Second, our study was conducted at a single health system so generalizability of our study results to other institutions must be cautioned; however, the six hospitals included were very distinct from each other. Third, patients who received antibiotics for concomitant infections including CAP were included within the study, which could bias toward recommendations for prolonged durations of therapy depending on the non-CAP infection being treated. Fourth, the study relied on patients’ admitting ED diagnoses, which might have been inaccurate and may have resulted in the unintended exclusion of eligible patients. Fifth, this study only assessed recommendations completed by a designated ID pharmacist and does not account for recommendations attempted by pharmacists on the rounding teams at the individual hospitals.

Overall, clinicians are receptive to ID pharmacist-led stewardship recommendations for CAP. However, more studies are needed to address concerns surrounding limiting the DOT. We identified significant opportunities to reduce the empiric use of ESAs for CAP and increase the shortest effective DOT aligning with current ASP guideline recommendations.

## Supporting information

Walker et al. supplementary material Walker et al. supplementary material

## Supplementary material

For supplementary material accompanying this paper visit http://doi.org/10.1017/ash.2024.399. click here to view supplementary material
