# Supplementary material for: Acceptance of pharmacist-led stewardship recommendations for patients with community-acquired pneumonia
Source: Antimicrob Steward Healthc Epidemiol. 2024 Oct 17;4(1):e181. doi: 10.1017/ash.2024.399 (PMC11500263; doi:10.1017/ash.2024.399)
Supplement: Walker et al. supplementary material [file S2732494X24003991sup001.docx]

Acceptance of Pharmacist-Led Stewardship Recommendations for Patients with Community-Acquired Pneumonia

**Ramara E. Walker, PharmD, BCIDP, AAHIVP,^1^** Rebecca Schulte, MPH,^2^ Andrea M. Pallotta, PharmD, BCPS, BCIDP, AAHIVP,^1^ Larisa G. Tereshchenko, MD, PhD,^2^ Victoria A. Criswell, BS,^3^ Abhishek Deshpande, MD, PhD,^3,4^ Michael B. Rothberg, MD, MPH^3^

Supplemental Material

Supplemental Material Legend

**E-Table 1**. Characteristics of Patients with and without a Recommendation (First Patient Encounter Only)

| **e-Table 1:** **Characteristics of Patients with and without a Recommendation (First Patient Encounter Only)** |  |
| --- | --- |

| **Characteristic** | **Overall**  **(N = 5,389)** | **No Recommendation**  **(N = 4,779)** | **Recommendation**  **(N = 610)** | ***P* Value** |
| --- | --- | --- | --- | --- |
| Age, years | 71 (16) | 71 (16) | 73 (15) | 0.005 |
| Gender |  |  |  | 0.6 |
| Female | 2,727 (51%) | 2,424 (51%) | 303 (50%) |  |
| Male | 2,662 (49%) | 2,355 (49%) | 307 (50%) |  |
| Ethnic Group |  |  |  | 0.5 |
| African American | 1,374 (25%) | 1,211 (25%) | 163 (27%) |  |
| Asian/Pacific Islander | 51 (0.9%) | 45 (0.9%) | 6 (1.0%) |  |
| Caucasian | 3,720 (69%) | 3,300 (69%) | 420 (69%) |  |
| Hispanic | 117 (2.2%) | 105 (2.2%) | 12 (2.0%) |  |
| Multi-Racial | 52 (1.0%) | 45 (0.9%) | 7 (1.1%) |  |
| Native American | 7 (0.1%) | 7 (0.1%) | 0 (0%) |  |
| Other | 50 (0.9%) | 49 (1.0%) | 1 (0.2%) |  |
| Patient Refused | 18 (0.3%) | 17 (0.4%) | 1 (0.2%) |  |
| Extended Spectrum Antibiotics | 2,700 (50%) | 2,369 (50%) | 331 (54%) | 0.029 |
| CURB65 score | 1 (1) | 1 (1) | 2 (1) | 0.12 |
| Severe CAP | 795 (15%) | 701 (15%) | 94 (15%) | 0.6 |
| Admission to ICU | 922 (17%) | 878 (18%) | 44 (7.2%) | <0.001 |
| Vasopressors | 416 (7.7%) | 394 (8.2%) | 22 (3.6%) | <0.001 |
| MRSA Swab | 2,718 (50%) | 2,393 (50%) | 325 (53%) | 0.14 |
| Positive MRSA Swab | 255 (9.4%) | 238 (9.9%) | 17 (5.2%) | 0.006 |
| Pneumococcal Vaccine | 4,139 (77%) | 3,664 (77%) | 475 (78%) | 0.5 |
| Respiratory Virus Panel | 625 (12%) | 542 (11%) | 83 (14%) | 0.1 |
| Positive Respiratory Virus Panel | 168 (27%) | 144 (27%) | 24 (29%) | 0.7 |
| Respiratory Culture | 1,276 (24%) | 1,105 (23%) | 171 (28%) | 0.007 |
| Positive Respiratory Culture | 391 (31%) | 341 (31%) | 50 (29%) | 0.7 |
| Blood Culture | 3,390 (63%) | 2,989 (63%) | 401 (66%) | 0.12 |
| Positive Blood Culture | 426 (13%) | 390 (13%) | 36 (9.0%) | 0.021 |
| Length of Stay, days | 5.7 (5.2) | 5.6 (5.2) | 6.7 (4.9) | <0.001 |
| Consult to Infectious Disease | 1,953 (36%) | 1,784 (37%) | 169 (28%) | <0.001 |
| Consult to Pulmonary Medicine | 1,850 (34%) | 1,596 (33%) | 254 (42%) | <0.001 |

1. Patient characteristic data are represented at the patient encounter level
2. Values are presented as n (%) or mean (SD) unless otherwise stated
3. Pearson’s Chi-squared test; Wilcoxon Rank Sum Test; Fisher’s Exact Test
